# Do Two Eyes Identify Better than One? Interpretable Binocular Dynamics for Virtual Reality Gaze Biometrics

**DOI:** 10.3390/jemr19040080

**Published:** 2026-07-22

**Authors:** Yiyu Zhang, Huijun Tang, Xiajing Yao

**Affiliations:** 1 School of Foreign Languages, China University of Geosciences (Wuhan), Wuhan 430074, China; yiyu.zhang@polyu.edu.hk (Y.Z.); tanghuijun@cug.edu.cn (H.T.); 2 Department of Language Science and Technology, The Hong Kong Polytechnic University, Hong Kong SAR, China

**Keywords:** gaze biometrics, virtual reality, vergence, fixation disparity, interocular coordination, continuous authentication, presentation attack detection

## Abstract

Virtual reality (VR) headsets with built-in eye trackers can record binocular gaze, but prior biometric studies have largely emphasized monocular gaze or general spatial trajectories, leaving the identity value of interocular coordination largely untested. We test whether that geometry provides identity information beyond monocular gaze dynamics. Using the GazeBaseVR dataset, we derive disparity, vergence-dynamic, and fixation disparity features and evaluate them in cross-session identification, task transfer, sequence modeling, replay detection, and return-visit analyses. Adding binocular features to monocular features improves cross-session Rank-1 identification, while binocular features alone provide complementary but limited discrimination. Performance is strongest when enrollment and probe tasks match and weakens sharply across tasks. Per-recording disparity centering leaves much of the binocular-only result intact, and a matched-input temporal convolutional network (TCN) performs better with binocular input than with duplicated monocular input. The TCN embedding overlaps with the disparity and vergence features, and simple feature-level fusion does not improve Rank-1 performance. Left-right gaze consistency can reject simple monocular replay attempts in this constrained setting, but score query adaptation reduces detection performance. In this setting, binocular dynamics are best treated as auxiliary evidence for VR gaze biometrics rather than as a standalone authenticator.

## 1. Introduction

Behavioral biometrics derived from eye movements combine the convenience of behavioral authentication with the physiological grounding of oculomotor control. Historically, this field evolved from analyzing basic reading scanpaths [1] and oculomotor plant models [2,3] to extracting complex, engineered features, such as fixation–saccade pattern descriptors [4], saccadic acceleration and vigor cues [5] and extensive feature catalogues [6]. More recently, the paradigm has shifted toward learned representations, where sequence models trained directly on gaze traces demonstrate strong verification performance [7,8]. As these approaches move into virtual reality (VR), recent studies have examined the biometric viability and data quality of headset-based recordings relative to high-end eye trackers [9], evaluated the long-term challenges of continuous authentication under template aging [10], and explored the fusion of learned verification models (e.g., Eye Know You Too) with continuous gaze-offset scores [11].

Crucially, binocular vision relies on coordinated oculomotor control. Several aspects of this coordination exhibit meaningful inter-individual variation and therefore offer potentially useful sources of biometric information. For instance, fixation disparity, the residual misalignment of the two visual axes during binocular fixation, can vary across observers [12,13], as does the coupling of accommodation and vergence, which is commonly parameterized by accommodative-convergence/accommodation (AC/A) and convergence-accommodation/convergence (CA/C) ratios [14,15]. Vergence includes an initial fast, preprogrammed component [16], and convergence is generally faster than divergence [17]. In the context of VR, integrated binocular eye trackers make it possible to estimate gaze disparity and vergence-related dynamics during head-mounted display (HMD) use [18,19,20]. Building on this capability, we derive interpretable binocular oculomotor features that characterize two-eye coordination, including disparity-based, vergence-dynamic, and interocular-consistency measures. We evaluate their cross-session verification utility and their complementarity with learned sequence models.

This study examines the extent to which binocular gaze improves identity verification in VR and identifies the information contributed by the second eye. We operationalize binocular recordings as estimates of gaze disparity, convergence and divergence, vergence excursion, and fixation disparity spectra. The present study is guided by three research questions (RQs):

- RQ1: Do interpretable binocular features add identity information beyond monocular gaze features?
- RQ2: How stable are the binocular signals across tasks, static disparity offsets, and return sessions?
- RQ3: Are binocular coordination patterns also captured by temporal convolutional network (TCN) sequence representations and left–right consistency cues for replay detection?

To address these questions, we organize the binocular measures into a unified oculomotor feature taxonomy and evaluate them using a predefined cross-session split of GazeBaseVR dataset [21]. Our protocol separates task transfer from static disparity offsets, compares feature-based models with matched-input TCNs, and evaluates left–right consistency across return sessions and under monocular replay attacks.

## 2. Related Work

### 2.1. Gaze Biometrics and Learned Eye Movement Representations

Behavioral biometrics derived from eye movements emerged as a credible identity modality in the early 2010s. Early approaches extracted subject-specific oculomotor signatures from reading scanpaths [1] and from fixation and saccade feature distributions [4]. Concurrently, a physiologically grounded line of research modeled identity using an oculomotor plant mathematical model (OPMM) to capture per-eye dynamics [2]. This OPMM framework was subsequently expanded to model oblique saccades on a 2D plane [22] and to evaluate the multi-session biometric efficacy of its internal anatomical parameters [23], alongside the formalization of the broader “Oculomotor Plant Characteristics” (OPC) feature family [3,24]. Beyond plant mechanics, saccadic vigor and acceleration were also shown to encode trait-like individuality [5,25], culminating in comprehensive engineered feature sets comprising over 100 descriptors for fixations, saccades, and post-saccadic oscillations [6].

A subsequent line of work shifted from hand-crafted descriptors toward end-to-end deep learning representations. A metric learning convolutional neural network (CNN) evaluated template aging on the longitudinal GazeBase dataset [7]. This was later advanced by a DenseNet-based temporal embedding model that approached Fast IDentity Online (FIDO) authentication targets (estimated 5% false rejection rate (FRR) at false acceptance rate (FAR) $10^{-4}$), though under statistically resampled evaluation constraints [8]. Following these models, studies have examined signal and noise across frequencies [26] and synthetic eye-tracking signal degradation [27]. Learned-embedding analyses have tested temporal persistence [28] and feature-to-embedding persistence [29]. Embedding reliability has also been evaluated as a performance predictor [30].

Efforts have also focused on enhancing model interpretability and hybridizing traditional physiological approaches, fusing continuous gaze offset scores with temporal embeddings [11], utilizing discrete gaze events as interpretable concepts for neural sequence models [31], and exploring alternative dynamic eye-movement features [32]. Concurrently, classical OPMM architectures have been modernized; recent formulations now estimate per-subject model reliability [33], cast plant mechanics in Kalman-filter forms for continuous gaze prediction [34], and analyze individual prediction variations across distinct eye-movement types [35].

Despite these advances, most existing research has focused primarily on monocular features. Traditional biometric approaches, whether by modeling reading scanpaths [1], fitting plant mechanics per eye [22], or aggregating large engineered feature sets [6], have largely relied on monocular information. This limitation also exists in modern deep learning frameworks; for instance, baseline DenseNet temporal embeddings report evaluations using only the left-eye recordings [8]. Furthermore, even when binocular VR recordings are natively available, recent VR biometric systems have commonly extracted identity from a single eye or from general spatial gaze trajectories [9]. While recent studies have incorporated joint left–right processing—such as four-channel binocular velocity models [36] or continuous gaze offset score fusions [11], existing frameworks have rarely treated interocular geometry itself as a primary and interpretable source of biometric information. In particular, dynamic disparity, vergence dynamics, and fluctuations in fixation disparity have received little direct study as explicit biometric features.

### 2.2. Binocular Vision, Vergence, and VR Eye-Tracking

Fixation disparity is a steady-state error of disparity-driven fusional vergence [12,37]. Evidence from prism adaptation studies shows that this error changes with vergence adaptation [38]. Disparity vergence shows preprogrammed motor control, with peak velocities following a main-sequence relationship [16,39]. Importantly, convergence and divergence are not mere mirror processes; they differ in time course, peak velocity, and dynamic asymmetry [17,40]. The coupling between accommodation and vergence can be studied through disparity- and blur-induced convergence responses [41], age-related relationships [14], and direct measurements of CA/C variation during stereoscopic viewing [15]. Age also can affect disparity vergence and phoria adaptation [42]. Research on individual differences in fixation disparity has examined the effects of viewing distance and prism load [43], as well as stimuli presented nearer or farther than oculomotor tonic positions [44]. Other work has documented child–adult variation in vergence dynamics [45] and compared objective and subjective fixation disparity under near-viewing and forced-vergence conditions [13,46]. Collectively, these findings indicate that binocular coordination is structured, dynamically regulated, and subject to measurable individual variation.

For biometric analysis, these quantities offer a distinct source of evidence, as they are measurable from binocular gaze traces, yet they describe interocular coordination rather than monocular event statistics or target-relative spatial offsets. VR exposure has been linked to changes in oculomotor function, including phoria, fixation disparity, AC/A ratio, stereopsis, and near point of convergence [18]. VR gaming studies have measured vergence dynamics under stereoscopic stimulation [19], and disparity tracking work has quantified coordinated vergence and pupil responses [20]. Methodological studies demonstrate that fixation disparity and binocular alignment can be quantified with explicit measurement protocols [47], and fusional reserves can be estimated with infrared eye tracking [48]. Saccade characteristics during fusional vergence tests vary with vergence demand and can be analyzed in normal binocular vision [49,50].

Clinical research also provides evidence that binocular coordination captures meaningful individual differences. Vergence endurance differs following concussion [51], eye alignment differs in Parkinson’s disease [52], and accommodation–vergence patterns vary across myopia-onset groups [53]. Fixation disparity measures have also been applied to children with strabismus [54]. At the same time, pupil-based binocular eye trackers require careful interpretation because pupil-size artifacts can inflate apparent vergence variability [55].

The availability of consumer HMDs with built-in eye trackers has made VR a practical setting for investigating gaze biometrics. Methodological work has demonstrated the feasibility of eye tracking within immersive VR environments [56], enabling applications spanning attention, foveated rendering, and gaze-based authentication [57,58]. Besides, behavioral biometrics extracted from controllers and head trajectories have also been studied [59,60]. To facilitate such research, GazeBaseVR [21] provides a 250 Hz binocular dataset of more than 5000 recordings from 407 participants tracked for up to 26 months, with five tasks including a dedicated vergence task.

VR biometric studies provide three useful baselines for the present work. Headset recordings can be compared with laboratory eye trackers [9], and recognition performance should be assessed over long return intervals [10]. Target-relative offset can also be fused with temporal embeddings to improve recognition performance [11].

### 2.3. Replay Attacks and Liveness in Gaze Authentication

Traditional start-of-session authentication leaves systems vulnerable to session hijacking, a motivation for continuous authentication noted in recent VR gaze biometric work [10]. Within this continuous paradigm, the principal attack surface is the gaze trace itself; an adversary who captures a target’s monocular trace in one session could potentially resynthesize or replay it to bypass verification in another. An early demonstration attacked an OPC system with mechanical replicas of the human eye, suggesting that plant characteristics can support liveness checks against physical replicas [61]. A related “counterfeit-resistant usable eye-movement authentication” (CUE) framework combined OPC with complex eye movement patterns to raise the cost of replay attacks [62]. Alongside these adversarial threats, robust biometric systems must also account for natural longitudinal failure modes, such as template aging, which intrinsically degrade authentication reliability over time [63].

As threat models evolve, recent research engages with more advanced attacks in VR. Motion forecasting attacks, for example, explicitly exploit the temporal structure of VR behavioral biometrics to degrade authentication accuracy [64]. Similarly, specialized datasets such as VRBiom introduce over 1,100 constructed presentation attacks for HMDs, including print attacks, fake 3D eyeballs, plastic eyes, and masks [65]. These modern evaluations are commonly framed using standardized presentation attack detection (PAD) terminology and protocols (ISO/IEC 30107) [66,67], and related PAD work also examines dynamic physiological cues such as pupil liveness for iris recognition [68]. In contrast, left-right gaze consistency, the geometric relationship between left and right eye dynamics, has received little direct study as a source of identity information in VR biometrics.

## 3. Materials and Methods

### 3.1. Dataset and Signal Processing

We evaluate our framework using GazeBaseVR [21], a 250 Hz binocular VR eye-tracking dataset collected via a modified HTC Vive equipped with a SensoMotoric Instruments (SMI) tethered eye tracker. The full dataset spans up to three recording rounds over a 26-month period. For our primary cross-session benchmark, we exclusively utilize round 1, as it is the only round where all 407 participants are present. Each participant contributes two sessions in this round, session 1 (S1) and session 2 (S2), recorded approximately 30 min apart. The smaller return cohorts from rounds 2 and 3 are reserved for the longitudinal analysis presented in Section 4.7. Five tasks are recorded in the dataset: vergence, smooth pursuit, video, reading, and random saccades.

Raw gaze directions were converted to binocular angular trajectories (in degrees), with horizontal and vertical components denoted by $L_{x,t},L_{y,t}$ for the left eye and $R_{x,t},R_{y,t}$ for the right eye. Each sample was represented as

(1) $$\begin{array}{cc} x_{t} & =(L_{x,t},L_{y,t},R_{x,t},R_{y,t},d_{x,t},d_{y,t},V_{t},m_{t}),\\ d_{x,t} & =L_{x,t}-R_{x,t},\;d_{y,t}=L_{y,t}-R_{y,t},\;V_{t}=\sqrt{d_{x,t}^{2}+d_{y,t}^{2}}, \end{array}$$

where $d_{x,t}$ and $d_{y,t}$ denote horizontal and vertical interocular angular disparity, respectively, and $V_{t}$ is their Euclidean magnitude, serving as a scalar representation of vergence-related binocular dynamics. The binary mask $m_{t}$ is a samplewise missingness indicator ($m_{t}=1$ when any raw eye channel is missing before interpolation and $m_{t}=0$ otherwise).

**Figure 1:**
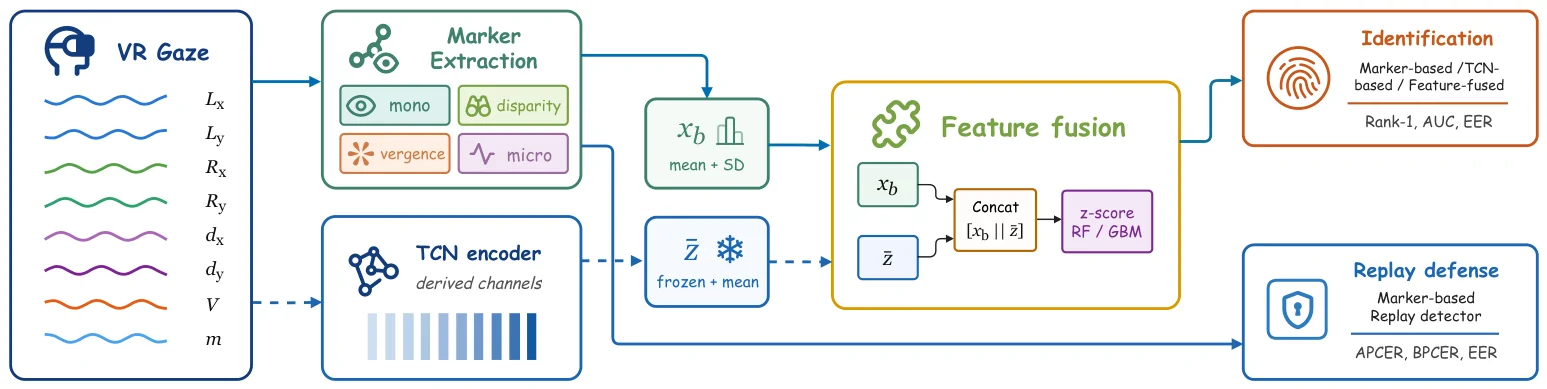
Overview of the study framework. Marker extraction provides features for identification, fusion, and replay detection. The vector $x_{b}$ summarizes marker features by mean and standard deviation, whereas TCN embeddings are mean-pooled into $\bar{z}$ and evaluated for identification alone or in fusion.

For the feature benchmark and TCN sequence comparison, we extract 5 s windows with a 2.5 s stride. The TCN sequence length analysis additionally incorporates 1, 5, and 10 s windows with a half-window stride. We retain windows with at least 50% valid samples after fellow-eye interpolation. Participants are partitioned by subject into training, validation, and test sets; Appendix A lists the split sizes and reproducibility settings. Figure 1 illustrates the framework of the study.

### 3.2. Binocular Oculomotor Features

To evaluate the added value of binocular coordination, our feature set separates conventional monocular gaze dynamics from quantities governed by the geometric relationship between the two eyes.

For the monocular baseline, we employ velocity-threshold identification (I–VT) with a 100 deg/s threshold to detect saccades. From these trajectories, we extract per-saccade amplitude, duration, and peak velocity, together with their distributional statistics, log–log main-sequence parameters (slope and intercept), and fixation duration and dispersion.

The proposed binocular features characterize interocular coordination at three complementary levels. First, disparity geometry captures the relative angular offset between the two eyes. We compute descriptive statistics, including means, standard deviations, percentiles, and temporal slopes, for horizontal ($d_{x}$) and vertical ($d_{y}$) disparity and for their Euclidean magnitude (*V*). This feature family also includes basic interocular descriptors, namely left–right speed asymmetry and spatial cross-correlation.

Second, vergence dynamics characterize how interocular disparity changes over time. These features quantify signed vergence velocity, identify convergence and divergence events, and summarize their peak velocity, duration, amplitude, and onset timing. We additionally derive total vergence excursion, excursion rate, and vergence–saccade coupling within a ±60 ms temporal window.

Third, spectral fixation disparity captures low-amplitude binocular fluctuations during stable fixations. Welch power spectral density (PSD) estimates of $d_{x}$, $d_{y}$, and *V* are computed over the 1–30 Hz band, from which we derive dominant frequency, band power, spectral entropy, the high-to-low frequency ratio, and drift variability.

**Figure 2:**
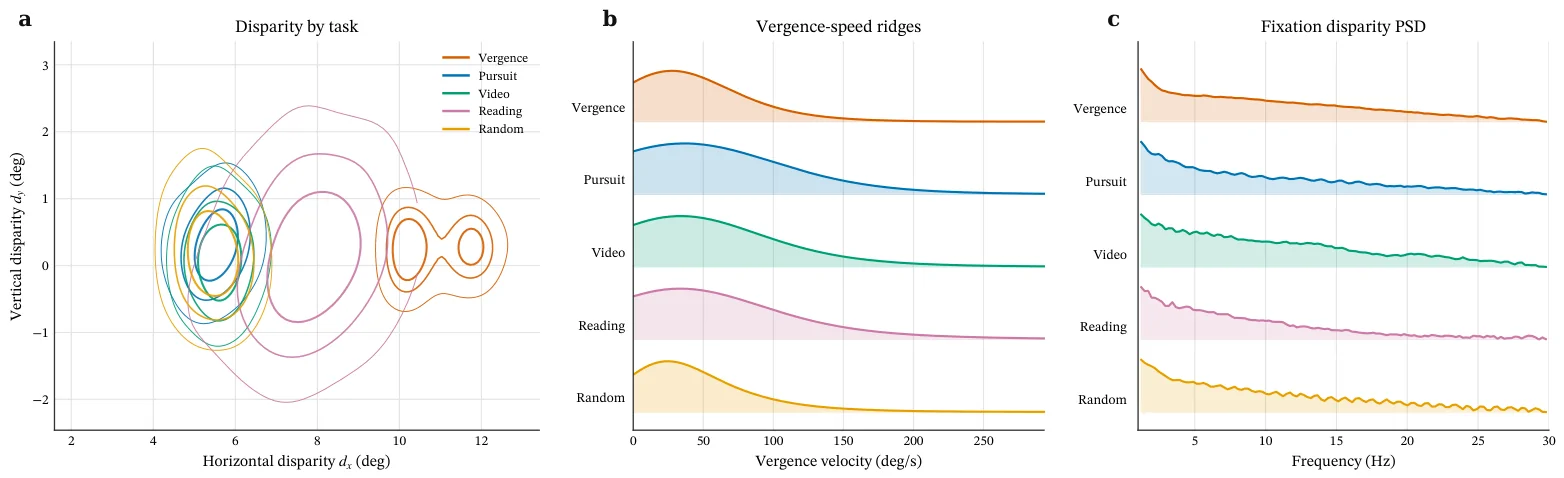
Signal distribution of the binocular features. (**a**) Kernel density contours of horizontal and vertical interocular disparity. (**b**) Density of signed vergence velocity. (**c**) PSD of fixation disparity in the 1–30 Hz band.

Detailed formulations for extracted features are provided in Appendix B. Figure 2 visualizes representative signal patterns across tasks, including disparity geometry, signed vergence-velocity distributions, and fixation disparity spectra.

### 3.3. Sequence Models and Fusion Strategies

To evaluate the discriminative power of the proposed metrics, our evaluation contrasts the monocular baseline, the binocular feature set, and their combination. We further conduct an ablation study on the combined set by iteratively removing disparity geometry, vergence dynamics, or spectral fixation disparity. For session-level evaluation, window-level feature vectors are aggregated into a single representation per recording (defined by subject, task, and session) by concatenating their temporal means and standard deviations.

To assess sensitivity to recording-level additive offsets, we apply zero-mean normalization to the horizontal and vertical disparities within each recording before feature extraction:

(2) $${\tilde{d}}_{x,t}=d_{x,t}-{\bar{d_{x}}}^{\left(r\right)},\;{\tilde{d}}_{y,t}=d_{y,t}-{\bar{d_{y}}}^{\left(r\right)},\;{\tilde{V}}_{t}=\sqrt{{\tilde{d}}_{x,t}^{2}+{\tilde{d}}_{y,t}^{2}}.$$

Here ${\bar{d_{x}}}^{\left(r\right)}$ and ${\bar{d_{y}}}^{\left(r\right)}$ denote recording-level means over retained preprocessed window samples from recording *r*. The centered magnitude ${\tilde{V}}_{t}$ is recomputed from the centered disparity vector. This operation subtracts a constant additive mean from each disparity axis. It attenuates constant offsets associated with headset placement or calibration, but it does not remove general IPD geometry, dynamic headset slippage, nonlinear calibration error, tracker noise, or device bias.

For the learned sequence evaluation, we train a supervised TCN for the 76 evaluation identities under the same closed-set session 1 (S1) to session 2 (S2) protocol. S1 windows are used for model fitting and enrollment, and S2 windows from the same identities form the probe set. The early-stopping split uses 20% of the S1 windows from these evaluation identities and is separate from the 76-participant cohort used for model selection. Prior binocular sequence models process four velocity streams from the left and right eyes [36]. To ensure a meaningful comparison and isolate the true physiological value of second eye information from the advantage of having more input channels, we therefore introduce a matched-dimensionality, monocular control. The four evaluated input configurations are

(3) $$\begin{array}{cccc} x_{\mathrm{left}} & =[L_{x},L_{y}], & x_{\mathrm{dup}} & =[L_{x},L_{y},L_{x},L_{y}],\\ x_{\mathrm{raw}} & =[L_{x},L_{y},R_{x},R_{y}], & x_{\mathrm{derived}} & =[L_{x},L_{y},R_{x},R_{y},d_{x},d_{y},V,m]. \end{array}$$

The matched input dimensionality comparison is repeated at 1, 5, and 10 s windows with half-window stride under the same subject partition.

And in order to investigate the complementarity of handcrafted markers and deep temporal representations, we construct fusion models. We freeze the TCN trained on the $x_{\mathrm{derived}}$ channels and extract sequence-level embeddings, aggregating them via mean-pooling. These pooled embeddings are then concatenated with the aggregated binocular feature vectors. We benchmark the TCN embeddings, the combined feature set, the fused representation, and ablated fusion variants to quantify the relative contribution of each feature family.

### 3.4. Evaluation Protocol

We evaluate feature and fusion models on standardized inputs using a Random Forest (RF) and a LightGBM gradient boosting model (GBM). The two classifiers have different roles. A validation rule, established prior to test evaluation, selects the RF as the primary performance benchmark for the primary feature evaluation. The GBM serves as a unified analytical model across all secondary evaluations, including task transfer, disparity centering, learned-sequence fusion, interpretability, and longitudinal analyses. Identification performance is reported using Rank-1 and Rank-5 accuracy; given our balanced probe set, Rank-1 is equivalent to balanced accuracy. For verification, the classifier’s posterior probability for each test sample and claimed identity pair serves as the match score, from which we derive the equal error rate (EER) and area under the receiver operating characteristic curve (AUC).

The primary feature protocol enrolls S1 and tests on S2 across sessions. We also evaluate a cross-task transfer matrix, wherein each individual S1 task is used for enrollment and each S2 task serves as the probe. TCN and fusion analyses utilize the cross-session protocol. For TCN models, window-level posteriors are averaged within subject, task, and session groups; feature-based models aggregate window-level inputs via mean and standard deviation. Because these aggregation strategies are tied to their respective computational paradigms, performance comparisons should be constrained to models within the same architecture. Confidence intervals and paired contrasts use subject-level bootstrapping with Holm correction for the predefined contrast families. Replicate counts, random seeds, and sensitivity checks are listed in Appendix A.

### 3.5. Monocular Replay Threat Model

Prior gaze biometric liveness studies have explored mechanical replicas of the eye [61] and counterfeit-resistant authentication based on oculomotor plant characteristics [62]. Motivated by these foundations, we formulate a monocular replay threat model. Specifically, we assume an attacker successfully intercepts a target’s left-eye trajectory during an enrollment session and attempts to authenticate during a subsequent session by synthetically generating the right-eye counterpart. In a naïve replay attack, the right eye trajectory is synthesized by simply applying the population mean disparity of the corresponding task to the captured left eye position:

(4) $${\hat{R}}_{t}=L_{t}-{\bar{d}}_{\mathrm{task}},\;L_{t}=\left(L_{x,t},L_{y,t}\right).$$

In the informed replay attack, the attacker substitutes a real impostor’s disparity trajectory for the matching task. Two further nonadaptive attacks probe more realistic threats. The perturbed target attack adds 1σ i.i.d. Gaussian noise to the target’s own disparity, and the transfer attack substitutes the same subject’s disparity from a different task. A binary GBM defender trained on disparity, vergence, and fixation disparity spectral features with disjoint subjects reports attack presentation classification error rate (APCER), bona fide presentation classification error rate (BPCER), EER, and AUC for each attack.

As none of these attacks adapt to the defender, we add an adaptive stress test with score queries. The attacker knows the deployed defender, a fixed model trained on real versus informed replay traces, and can query its bona fide scores. Because the GBM is not differentiable, the attack uses black-box 1+λ search (approximately 64 queries per window) to maximize this score over a low-dimensional synthesis of $d=(d_{x},d_{y})$: per-axis affine transformations of an impostor trajectory plus the first three low-frequency cosine modes. At each time point, the synthesized disparity is clipped to the task-specific genuine envelope $\left[p_{1},p_{99}\right]$, constraining the resulting fellow-eye trajectory to the empirical range observed in genuine recordings.

## 4. Results

### 4.1. Identity Information in Binocular Features

We first report the primary feature benchmark. Both classifiers ranked the feature sets identically for identification, with the combined monocular–binocular representation performing best (Table 1). This table provides the comparison between Random Forest and GBM; all subsequent explanatory analyses use GBM as the fixed analytical model.

In the S1→S2 subject-aggregate analysis, every feature set is above the 76-way chance baseline. For the headline RF, Rank-1 rises from 0.213 for monocular features to 0.255 for binocular features alone and 0.295 for the combined set; EER and AUC follow the same monotonic ordering. The GBM reproduces the same Rank-1 ordering but is less consistent on the verification metrics. Paired contrasts give the sharper test. For the headline RF, combined minus monocular Rank-1 is +0.081, 95% confidence interval (CI) [0.039,0.126], and Rank-5 is +0.097, [0.042,0.147], with both p<0.001 after Holm correction. The combined versus monocular Rank-1 gain is likewise significant for the GBM, at +0.108, [0.055,0.163]. The binocular-only and verification contrasts remain suggestive but unresolved after correction.

Session-level grouping makes the combined advantage most visible. The combined feature set reaches the highest grouped Rank-1, 0.397 [0.328, 0.461], numerically above the monocular set at 0.318 [0.263, 0.371] and the binocular set alone at 0.308 [0.241, 0.379]. This is the largest separation we observe between feature sets, but under subject-level resampling the three intervals overlap. The higher grouped Rank-1 reflects an easier session decision and should be read separately from the subject-aggregate result.

Because all included windows already satisfy a minimum valid sample fraction, we also tested whether the main pattern depends on lower validity windows. The quality robustness analysis reruns the feature benchmark after retaining only windows with valid fractions of at least 0.50, 0.90, and 0.95 across both classifiers, the subject-aggregate and window-level protocols, and the task-holdout split (Appendix C, Table A3 and Table A4). All three filters retain all 76 test identities. In the primary S1→S2 benchmark, the combined feature set remains strongest at the stricter thresholds for both classifiers: RF combined Rank-1/AUC is 0.305/0.890 at ≥0.90 and 0.300/0.885 at ≥0.95, while GBM combined Rank-1/AUC is 0.247/0.874 and 0.255/0.869. Thus, the measurable binocular contribution is not driven by the lowest-validity windows.

### 4.2. Task Dependence and Static Disparity Offsets

**Figure 3:**
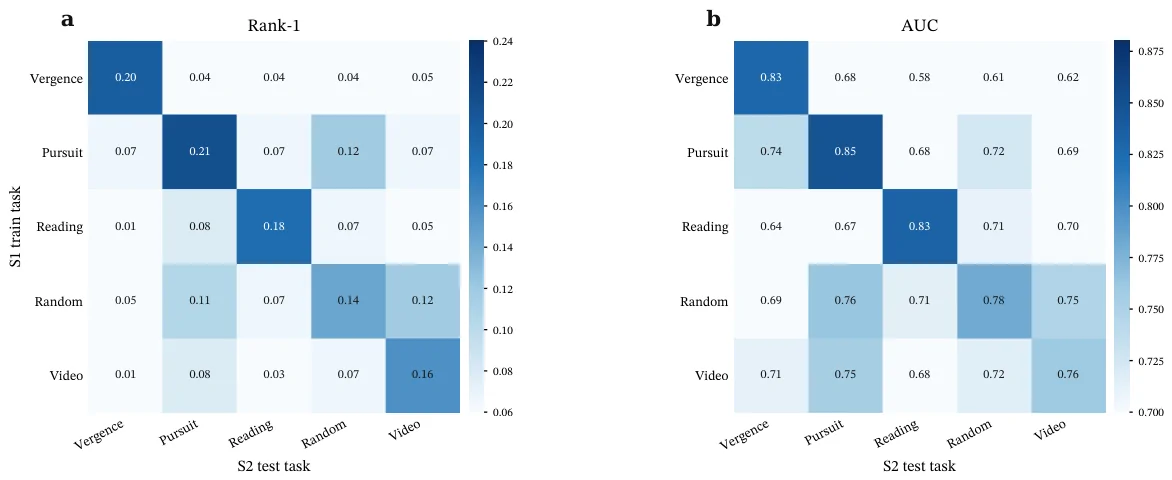
Task-transfer matrix for the combined feature set. (**a**) Rank-1 identification. (**b**) AUC. Each cell trains on one S1 task and tests on one S2 task for the 76 test subjects.

Biometric discriminability varies markedly across visual tasks. Smooth pursuit and the purpose-designed vergence task yield the strongest identification, with Rank-1 values of 0.211 and 0.197, while random saccades is the weakest at 0.145. To investigate this variability, we construct a cross-task transfer matrix (Figure 3).

The matrix highlights this task dependence. For the combined feature set, cells on the diagonal demonstrate superior performance, averaging a Rank-1 of 0.179 and an AUC of 0.810. In contrast, cross-task transfer (off-diagonal elements) severely degrades performance, dropping the averages to Rank-1 0.061 and AUC 0.691, with most off-diagonal Rank-1 values lingering near the 76-way chance baseline. The degradation persists across both classifiers and under valid-sample filters (Appendix C, Table A5).

Beyond classification performance, we assess the temporal persistence of these features using the intraclass correlation coefficient, ICC(1,1), which has been linked to biometric classification performance [69]. The summaries are in Appendix C, Table A6. Averaged across the five tasks for each subject’s two S1 sessions, monocular and vergence features demonstrate moderate reliability (mean ICC of 0.56 and 0.55, respectively). Conversely, absolute disparity features exhibit the lowest stability (ICC = 0.30), consistent with session-to-session instability, measurement noise, calibration variation, and headset placement changes.

To assess sensitivity to recording-level additive offsets, we evaluate the impact of disparity centering (Table 2). After centering, standalone binocular features retain a Rank-1 accuracy of 0.213 and an AUC of 0.846, essentially preserving the raw baseline performance (ΔRank-1 = −0.013, 95% CI [−0.053,0.029]). For the combined feature set, centering induces a marginal decrease in Rank-1 accuracy (from 0.274 to 0.232; Δ=−0.043, 95% CI [−0.082,−0.003]), yet preserves overall discriminative power, with the AUC remaining practically identical (0.869 vs. 0.870). These results indicate that recording-level additive offsets provide a minor boost to closed-set identification rankings, but that a measurable binocular signal remains after those offsets are attenuated.

### 4.3. Learned Sequence Comparison

We summarize the learned sequence and fusion levels alongside the feature benchmark in Table 3. The TCN rows use Rank-1 with 95% bootstrap intervals and AUC after averaging window posteriors within each subject, task, and session group. Identification across sessions rises from the left-eye view, Rank-1 0.087 [0.049, 0.132], to the raw binocular view, 0.187 [0.135, 0.238]. The paired difference test confirms this jump as significant. Raw binocular minus left eye Rank-1 is +0.101, 95% CI [0.050,0.155], with AUC moving in step and both Holm-corrected p<0.001. Adding derived disparity and vergence channels takes Rank-1 a further step to 0.216 [0.157, 0.283]; AUC is 0.749, 0.827, and 0.854 across the three views. The additional gain over raw binocular input was not statistically resolved after Holm correction.

The monocular control with matched input dimensionality separates binocular information from input dimensionality. Duplicating the left eye to four channels performs essentially identically to the genuine left eye view. Rank-1 is 0.089 versus 0.087, with paired difference +0.003 and 95% CI [−0.026,0.032]. Raw binocular input significantly outperforms the duplicated left eye comparator, with Rank-1 difference +0.098, [0.050,0.153], and AUC difference +0.072, [0.036,0.107]; both Holm-corrected p<0.001.

**Figure 4:**
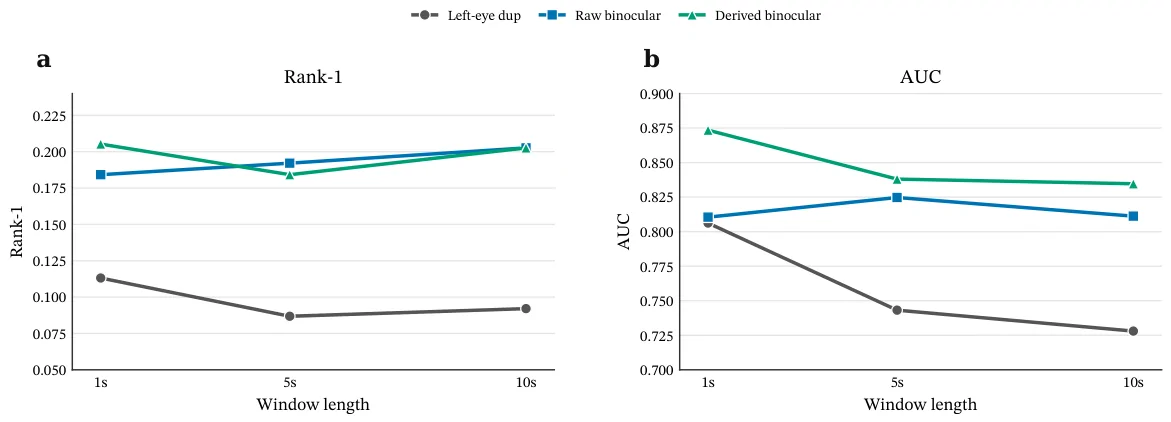
Observation window length comparison for the TCN. (**a**) Rank-1 across 1, 5, and 10 s windows. (**b**) AUC across the same windows.

The input check covers 1, 5, and 10 s window lengths to test whether the raw binocular advantage changes (Figure 4). Raw binocular input improves Rank-1 over the duplicated left eye comparator at all three lengths by +0.071, +0.106, and +0.111, and each paired bootstrap interval excludes zero. The AUC difference is positive at all lengths and resolved at 5 and 10 s; at 1 s the raw versus duplicate AUC gap is small, while the derived binocular channels lift AUC to 0.873.

Fusing the TCN embedding with the binocular features does not improve on the features alone. The combined feature set reaches Rank-1 0.274 [0.205, 0.339], above the fused model at 0.247 [0.192, 0.315] and the embedding alone at 0.211 [0.153, 0.272]. Fusion AUC is 0.873, nearly identical to the feature-set AUC of 0.869. Among the family removal analyses (Appendix C, Table A2), removing vergence lowers fusion AUC most, from 0.873 to 0.842.

### 4.4. Binocular Feature Attribution

**Figure 5:**
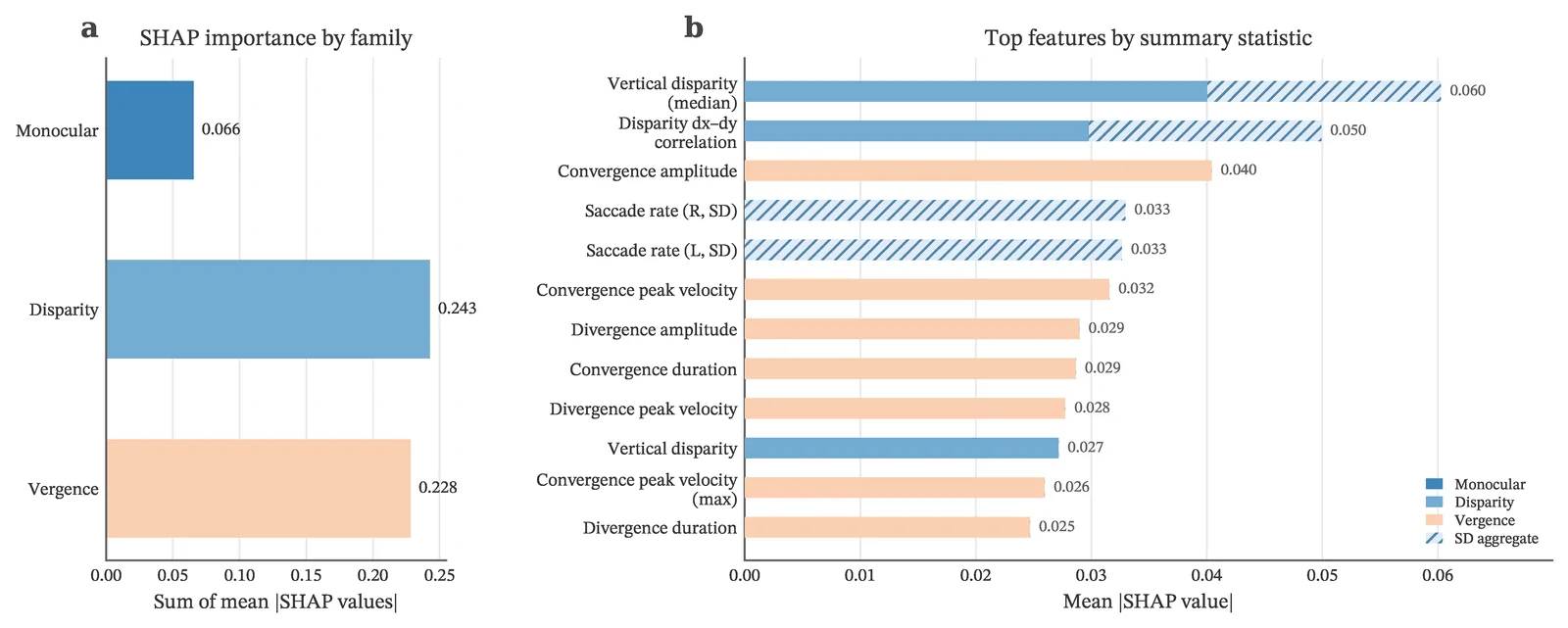
SHAP attributions for the combined feature classifier. (**a**) Sum of the mean absolute SHAP values for the top 20 features, grouped by feature family. (**b**) Mean absolute SHAP values for the top features, separated into window mean and standard deviation aggregates. Bar colors indicate feature family, and hatching indicates standard deviation aggregates.

Shapley additive explanations (SHAP) analysis of the GBM classifier reveals that vergence dynamics, specifically convergence and divergence amplitude, peak velocity, and duration, dominate the model’s attributions (Figure 5). Interocular disparity also ranks highly, driven by horizontal and vertical disparity statistics alongside the $d_{x}$–$d_{y}$ correlation, paired with monocular saccade rates. Notably, the top-ranking predictors comprise a mix of window means and standard deviation aggregates. For instance, vergence amplitudes and peak velocities primarily contribute through their typical magnitudes (means), whereas saccade rates and various disparity spread descriptors contribute via their within-recording variability (standard deviations). In contrast, no fixation disparity spectral descriptors emerge among the highest attribution features. The random forest and the GBM align closely in their top feature attributions, both relying heavily on vergence and disparity. While these SHAP values elucidate the classifier’s statistical dependencies within this specific representation space, they should be interpreted as predictive correlates.

### 4.5. Interpreting Learned Binocular Representations

**Figure 6:**
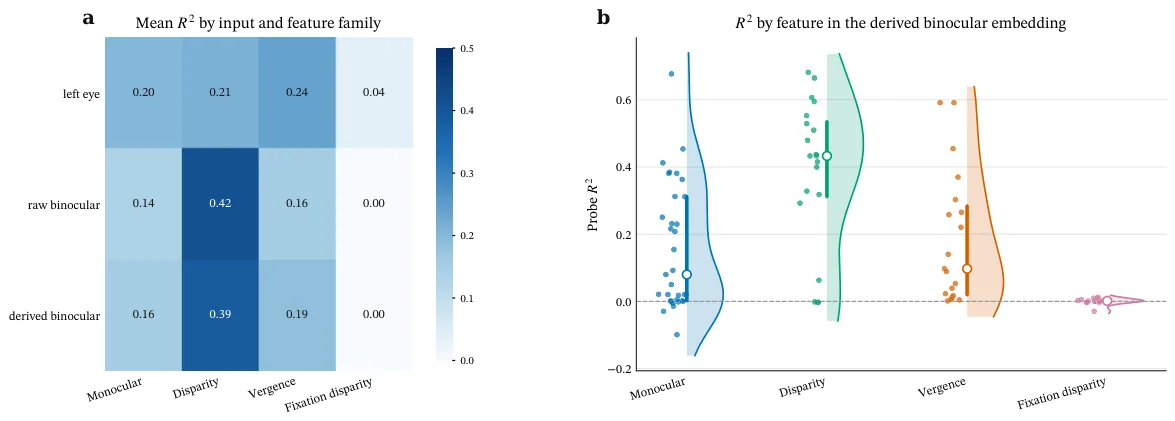
Linear probes of frozen TCN embeddings. (**a**) Mean $R^{2}$ for each input view and feature family, using splits defined at the subject level. (**b**) $R^{2}$ values for individual features predicted from the derived binocular embedding. Half violins show kernel density estimates, points show individual features, vertical bars show interquartile ranges, and open circles show medians.

Linear probes test whether TCN embeddings retain those same interpretable signals. For each TCN variant, we fit a RidgeCV model to predict individual window’s features from the embedding under splits by subject. Figure 6 summarizes the resulting feature-family and per-feature $R^{2}$ values. We report feature family mean $R^{2}$ values in Appendix D, Table A7. Shuffling embedding–feature pairings yielded $R^{2}\approx 0$, confirming that the recovered structure exceeded chance.

The probes showed selective recovery of marker information. Disparity features were substantially more predictable from raw- and derived-binocular embeddings than from the left-eye embedding, consistent with the absence of direct interocular information in the monocular input. In contrast, fixation disparity spectral features remained near zero across embeddings. Together with the SHAP and family removal analyses, this indicates that spectral markers made little measurable contribution to identity prediction in this dataset. The recoverability of disparity features is also consistent with the limited benefit of feature fusion, as the TCN embedding and marker set encode overlapping binocular structure.

### 4.6. Monocular Replay Detection

**Figure 7:**
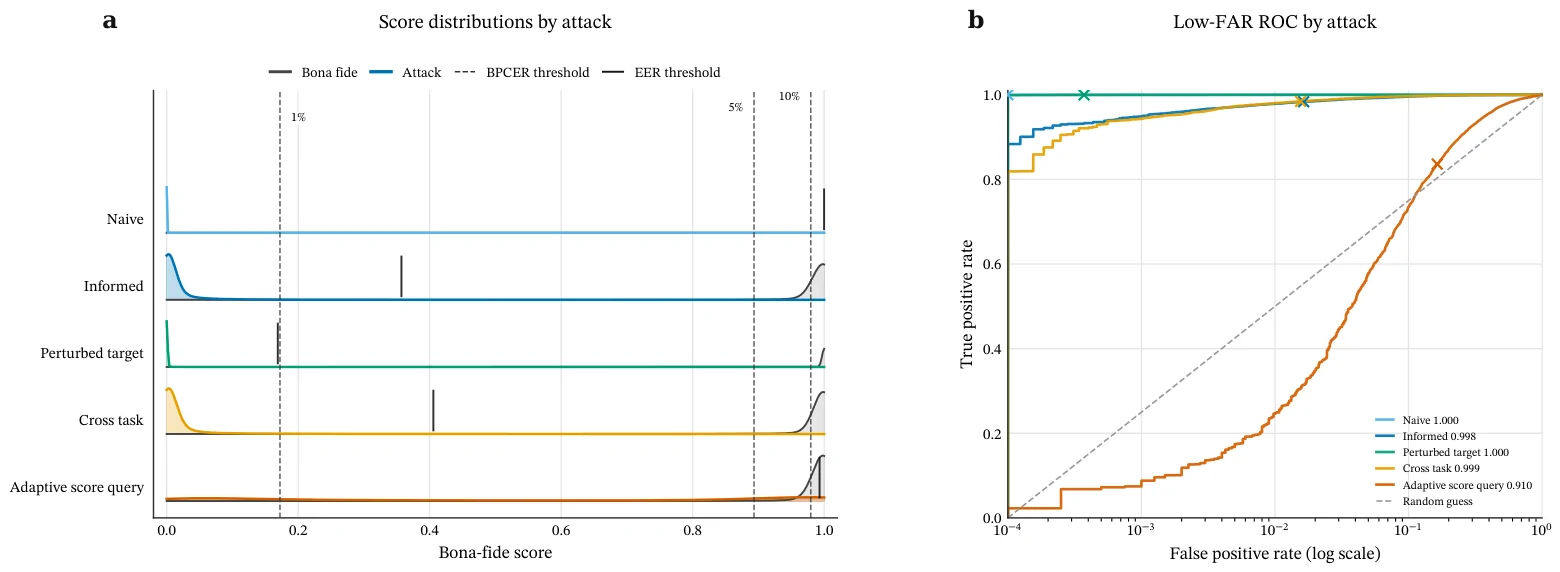
Replay-detection scores and receiver operating characteristic (ROC) curves for the left–right consistency defender. (**a**) Bona fide and attack score distributions with BPCER and EER thresholds. (**b**) Per-attack ROC curves on a false positive rate axis; × marks EER points and the dashed line indicates random guessing performance.

As summarized in Table 4 and visualized in Figure 7, population, impostor, perturbed target, and task-transfer disparity attacks are almost completely rejected by the left-right consistency defender. This result is preliminary evidence that simple monocular replay leaves detectable left-right inconsistencies, not a mature liveness defense. The adaptive score query attack changes the picture. With a low-dimensional trajectory parameterization and roughly 64 queries per window, many spoof traces move into the bona fide score region.

### 4.7. Longitudinal Stability

**Figure 8:**
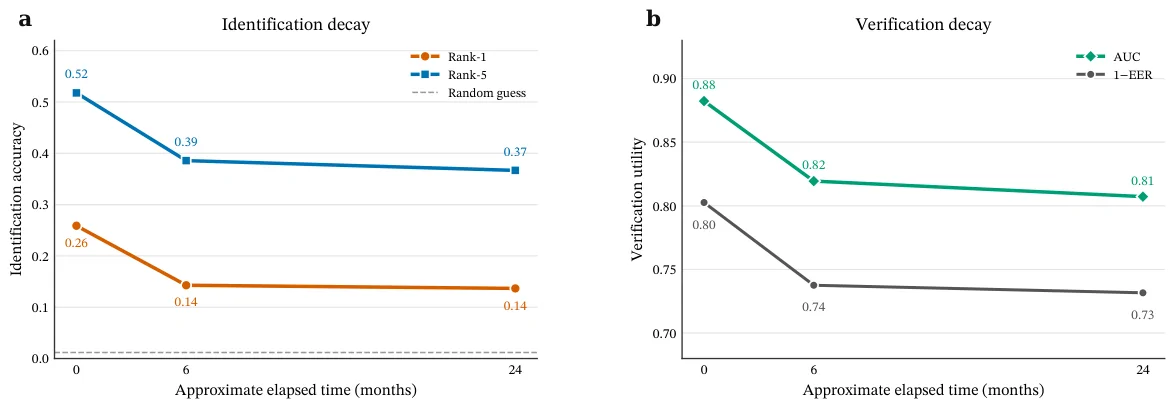
Longitudinal generalization on an approximate elapsed time axis. (**a**) Rank-1 and Rank-5 identification of the round 1 gallery at the round-1 S1→S2 baseline, approximately 6-month, and approximately 24-month probes; the dashed line marks the 85-way random guess baseline. (**b**) Verification utility over the same intervals as AUC and 1−EER. Round 2 and round 3 use different return cohorts.

The main benchmark uses the two round-1 sessions separated by approximately 30 min; the later rounds from the dataset allow us to examine how feature-based identification holds up as time passes. For this longitudinal check, a round 1 gallery from the 85 participants who returned for a later round is used to identify their round 2 and round 3 recordings in a closed-set 85-way task (Table 5; Figure 8). Round 2 occurs months later, and round 3 extends up to roughly two years later. Rank-1 falls from 0.259 at the round-1 S1→S2 baseline to 0.143 at round 2 and 0.137 at round 3; AUC declines from 0.882 to 0.819 and 0.807. This drop is consistent with prior VR gaze biometric results over a 26-month span [10]. The round 2 and round 3 probe cohorts overlap in only ten participants, so the two later points are not a single fixed cohort. The round 3 Rank-1 remains above the 1/85 chance level but is far below practical authentication needs without renewal, fusion, or other safeguards.

## 5. Discussion

Our evaluations indicate that binocular information can serve as a complementary biometric cue. While the addition of binocular features improves Rank-1 accuracy to 0.295 in the 76-subject closed-set evaluation and reaches 0.143 in the longitudinal return cohort, these absolute performance levels remain below the strict thresholds required for primary authentication. Nevertheless, these signals may have practical value for continuous risk scoring, presentation attack screening, and multimodal zero-trust architectures that fuse gaze with head kinematics, controller telemetry, and conventional credentials. Therefore, the statistically significant performance gains show measurable supplementary identity information rather than standalone operational sufficiency.

Task dependence constitutes a central operational boundary for this modality. Matched tasks preserve significantly more identity information than cross-task transfers, primarily because distinct activities, such as pursuit, reading, and targeted vergence, have different oculomotor patterns. This behavioral variability limits seamless, naturalistic VR authentication where the user’s immediate visual task is uncontrolled or unknown. This interpretation is also compatible with broader eye-movement modeling evidence that task context can change measured gaze patterns [70], although that evidence comes from translation rather than VR authentication. Deploying such systems in practice will therefore necessitate fixed short challenge-response tasks, multi-context enrollment protocols, task-conditioned score calibration, or domain adaptation techniques.

Our explanatory analyses identify likely sources of the observed biometric signal. The TCN control indicates that the gain from binocular input is not merely a consequence of increased input dimensionality. Furthermore, the per-recording centering analysis suggests that while recording-level additive offsets slightly aid closed-set identification, a binocular signal remains after those offsets are attenuated. Finally, linear probing shows that the latent TCN embeddings encode disparity geometry, which helps explain why fusion of handcrafted features yields diminishing returns.

Beyond identity verification, binocular dynamics may also support replay screening by testing whether a submitted trace preserves plausible left-right consistency. The proposed mechanism rejects non-adaptive replays in our experiments, but this should be interpreted as an initial stress test against a baseline threat model. Our adaptive score-query attack raised the EER to 16.4% by optimizing plausible disparity trajectories. Consequently, a larger query budget, a more sophisticated trajectory generator, or the simultaneous compromise of both eyes would likely degrade this defense further. Securing biometric deployments will ultimately require strict query rate limits, reduced score feedback, randomized challenge tasks, and the integration of multi-layered liveness safeguards.

## 6. Limitations

The dataset is large by oculomotor biometric standards but remains modest compared with face or iris benchmarks. Moreover, the use of a fixed test cohort means that the reported uncertainty estimates capture within-cohort variability, but not the variability that might result from different partitions of the 407 round-1 participants.

Hardware limitations also impose certain constraints. The 250 Hz sampling rate is sufficient for the disparity, vergence-event, and 1–30 Hz spectral summaries used in this study, but it limits finer-grained analyses of short latency binocular events and higher frequency fixational dynamics. This limitation is most relevant to the fixation disparity spectral features. In the family removal, SHAP, and embedding probe analyses, these descriptors contributed little or no net identity information. This null contribution should therefore be interpreted cautiously, as higher sampling rates, lower tracker noise, more precise fixation segmentation, or alternative spectral summaries may be needed to evaluate this feature family more fairly.

Signal quality is another important aspect. The robustness analysis shows that the main ordering is stable after excluding windows below 0.90 and 0.95 valid fraction across classifiers, aggregation protocols, split modes, and task-transfer summaries (Appendix C, Table A3, Table A4, Table A5), but valid fraction is only a missingness proxy. It does not directly measure calibration quality, headset fit, IPD estimation error, headset slippage, tracker noise, or device biases. The vergence and disparity features are computed from the gaze direction geometry in Equation 1, not from pupil size estimation. Pupil-based binocular eye trackers can show apparent vergence variability driven by pupil size artifacts [55]; that specific artifact does not directly apply to our definitions, though pupil-dependent tracking error, IPD fit, headset slippage, and calibration drift may still affect the disparity channels. Generalization across headset models and eye-tracking pipelines, therefore, remains to be tested. The vergence excursion rate is inferred from stimulus-linked vergence dynamics, so it should be interpreted only as a descriptive marker.

There are methodological bounds in our modeling and security evaluations. The TCN results compare input views under one fixed training. The longitudinal analysis rests on small return cohorts, with 35 round 2 and 60 round 3 participants, and only 10 participants appear in all three rounds. The replay threat model assumes a clean captured monocular trace, and the adaptive attack is deliberately constrained to a low-dimensional parameterization with a modest query budget. Stronger generative attacks, or attacks that compromise both eyes simultaneously, remain outside the scope of this defense.

## 7. Conclusions

Two-eye coordination serves as a complementary biometric modality in VR environments. The discriminative value of binocular dynamics is supported by their persistence after recording-level disparity centering and by matched-dimensionality sequence controls. Nevertheless, the operational envelope of these signals is bounded by task dependence and longitudinal degradation. From a security perspective, left-right physiological consistency can help reject simple non-adaptive monocular replay attacks, yet the system remains vulnerable to adaptive, black-box score optimization. Realizing the full potential of binocular biometrics will require future research to prioritize task-conditioned authentication protocols, cross-device validation at higher sampling frequencies, and the development of countermeasures against high-fidelity generative attacks.

## Figures and Tables

**Table 1:** Feature-based identification and verification across sessions on the test set. Random Forest was selected on the validation set as the prespecified primary benchmark, whereas gradient boosting was used for secondary analyses. With balanced probes, Rank-1 accuracy equals balanced accuracy.

Feature Set	Rank-1	Rank-5	EER	AUC
Random Forest				
Monocular features	0.213	0.495	0.218	0.870
Binocular features	0.255	0.568	0.197	0.890
Mono + binocular features	0.295	0.592	0.189	0.892
Gradient boosting				
Monocular features	0.166	0.476	0.218	0.861
Binocular features	0.232	0.495	0.234	0.844
Mono + binocular features	0.274	0.539	0.208	0.869

**Table 2:** Per-recording disparity centering analysis. The centered condition subtracts each recording’s mean horizontal and vertical disparity before feature extraction, then recomputes vergence magnitude. Delta columns report the paired subject-bootstrap mean for centered minus raw.

Feature Set	Raw Rank-1/AUC	Centered Rank-1/AUC	ΔRank-1	ΔAUC
Binocular features	0.226/0.844	0.213/0.846	−0.013	0.003
Mono + binocular features	0.274/0.869	0.232/0.870	−0.043	0.001

**Table 3:** S1→S2 performance for feature, TCN, and fusion models. Feature or fusion models use GBM with mean–SD summaries, whereas TCN models average window posteriors by subject, task, and session. The 4-channel control duplicates the left eye.

Level	Model	Rank-1	Rank-5	EER	AUC
Feature	Monocular features	0.166	0.476	0.218	0.861
Feature	Binocular features	0.232	0.495	0.234	0.844
Feature	Mono + binocular features	0.274	0.539	0.208	0.869
TCN	TCN left eye	0.087	0.274	0.329	0.749
TCN	TCN left eye, duplicated 4ch control	0.089	0.287	0.318	0.760
TCN	TCN raw binocular	0.187	0.442	0.245	0.827
TCN	TCN derived binocular	0.216	0.466	0.234	0.854
Fusion	Fusion, TCN + binocular features	0.247	0.553	0.216	0.873

**Table 4:** Replay-attack performance of the left–right consistency defender. APCER is reported at fixed BPCER operating points of 1%, 5%, and 10%; EER and AUC are included for reference.

Attack	EER	APCER@1%	APCER@5%	APCER@10%	AUC
Naive replay	0.000	0.000	0.000	0.000	1.000
Informed replay	0.016	0.035	0.001	0.000	0.998
Perturbed target	0.000	0.000	0.000	0.000	1.000
Transfer across tasks	0.016	0.030	0.001	0.000	0.999
Adaptive score query	0.164	0.687	0.373	0.243	0.910

**Table 5:** Longitudinal generalization. A round 1 enrollment gallery of the returning participants is identified at increasing elapsed times. The comparison uses a round 1 S1→S2 baseline separated by approximately 30 min, then round 2 probes months later and round 3 probes up to ∼2 years later as a closed-set *N*-way task with oculomotor features.

Elapsed	*N*-Way	Probe Subjects	Rank-1	Rank-5	AUC
Round 1 S1→S2 baseline (∼30 min)	85	85	0.259	0.518	0.882
Round 1→Round 2, ∼months	85	35	0.143	0.386	0.819
Round 1→Round 3, up to ∼2y	85	60	0.137	0.367	0.807

## Data Availability

The dataset used in this study is publicly available from the GazeBaseVR figshare repository at https://doi.org/10.6084/m9.figshare.21308391 (accessed on 17 May 2026) and was originally introduced in Lohr et al. (2023) [21].

## Abbreviations

The following abbreviations are used in this manuscript:

AC/A	Accommodative-convergence/accommodation ratio
APCER	Attack presentation classification error rate
AUC	Area under the receiver operating characteristic curve
BPCER	Bona fide presentation classification error rate
CA/C	Convergence-accommodation/convergence ratio
CNN	Convolutional neural network
CUE	Counterfeit-resistant usable eye-movement authentication
EER	Equal error rate
GBM	Gradient-boosted tree model
ICC	Intraclass correlation coefficient
IPD	Interpupillary distance
I-VT	Velocity-threshold identification
OPC	Oculomotor plant characteristics
OPMM	Oculomotor plant mathematical model
PSD	Power spectral density
SHAP	Shapley additive explanations
TCN	Temporal convolutional network

## Appendix A. Reproducibility Details

Round-1 participants were partitioned once, using seed 20260517, into 255 development, 76 validation, and 76 evaluation participants. The validation cohort was used for model and hyperparameter selection. The primary estimates use a closed-set protocol on the 76 evaluation identities: we fit models or form enrollment representations from their S1 recordings and evaluate them on S2 recordings from the same identities. The development and validation participants are not included in these primary test estimates. This design measures cross-session performance for enrolled identities; it does not test recognition of identities absent from model fitting or enrollment. Random Forest was selected as the primary classifier because it had the higher mean validation AUC across the three primary feature sets on the S1→S2 split, 0.872 versus 0.853 for GBM. GBM is then fixed for the secondary analyses.

The Random Forest uses 400 trees, unbounded depth, and minimum leaf size 2. The GBM uses LightGBM 4.6.0 with 400 estimators, learning rate 0.04, 15 leaves, minimum leaf size 5, row subsample 0.6, column subsample 0.4, and an $L_{2}$ penalty of 2.0. The same seed controls classifier seeds, TCN initialization and batch shuffling, and bootstrap resampling unless a sensitivity check varies the training seed. The TCN early-stopping split uses 20% of S1 windows within the 76 evaluation identities. TCN grouped metrics average window posteriors within subject, task, and session groups, giving 380 test groups. Bootstrap intervals use 500 subject-level replicates; paired contrasts use 2000 paired subject-level replicates with Holm correction within each analysis family. The TCN comparator’s architecture and training budget are in Table A8; the feature families and counts are in Table A1, with formulas in Appendix B.

## Appendix B. Feature Catalogue and Computation Details

**Table A1:** Oculomotor feature families. Window-level feature vectors have 88 dimensions, with 33 monocular, 20 disparity, 19 vergence, and 16 fixation disparity spectral features. Aggregation by subject-level mean and standard deviation doubles this to 176. The four families are the basis for the GBM and Random Forest feature classifiers and the family removal analysis.

Family	Description	Dims
Monocular	Saccade and fixation statistics for each eye, plus L/R correlation	33
Disparity	Interocular position statistics for d*_x_* and d*_y_*	20
Vergence dynamics	Event count, excursion proxy, coupling	19
Fixation disparity spectral	Power spectral density descriptors in 1–30 Hz band	16
Window total		88
Subject total, mean + std		176

The features are computed per window at the dataset sampling rate $f_{s}$, on the preprocessed channels $\left(L_{x},L_{y},R_{x},R_{y},d_{x},d_{y},V,m\right)$.

**Angular speed and saccades.** Per-eye/cyclopean angular speed is computed from consecutive position differences:
  (A1) $$s_{t}=∥\left(\Delta x_{t},\Delta y_{t}\right)∥f_{s}.$$
  Saccades are I-VT events where $s_{t}>100$ deg/s for at least 3 samples. Each event’s amplitude, duration, and peak velocity are
  (A2) $$A=∥\left(x_{\mathrm{post}}-x_{\mathrm{pre}},y_{\mathrm{post}}-y_{\mathrm{pre}}\right)∥,\;D=\frac{t_{\mathrm{stop}}-t_{\mathrm{start}}}{f_{s}},\;v_{\mathrm{peak}}=\max_{t\in \mathrm{event}}s_{t}.$$
  The main-sequence descriptors are the slope and intercept of a degree-1 fit of $\log(v_{\mathrm{peak}})$ on log(A).
**Disparity and vergence.** Horizontal and vertical disparity and vergence magnitude are
  (A3) $$d_{x}=L_{x}-R_{x},\;d_{y}=L_{y}-R_{y},\;V=\sqrt{d_{x}^{2}+d_{y}^{2}}.$$
  Disparity features are the means, standard deviations, percentiles, slopes, magnitude $∥\left(d_{x},d_{y}\right)∥$, and the $d_{x}$–$d_{y}$ correlation.
**Convergence/divergence events.** On the signed disparity velocity
  (A4) $${\dot{d}}_{x,t}=\left(d_{x,t}-d_{x,t-1}\right)f_{s},$$
  restricted to non-saccadic samples, defined as cyclopean speed <30 deg/s, a convergence event is a run with ${\dot{d}}_{x}<-15$ deg/s and a divergence event a run with ${\dot{d}}_{x}>+15$ deg/s. The event descriptors are event count, peak $|{\dot{d}}_{x}|$, duration, absolute amplitude $|d_{x}\left[t_{\mathrm{end}}\right]-d_{x}\left[t_{\mathrm{start}}\right]|$, where $t_{\mathrm{end}}$ is the final in-run sample, and onset latency, defined as the time of the first $|{\dot{V}}_{t}|>15$ deg/s crossing with ${\dot{V}}_{t}=\left(V_{t}-V_{t-1}\right)f_{s}$.
**Vergence–saccade coupling.** For each saccade run with first and final in-event samples $t_{\mathrm{start}}$ and $t_{\mathrm{end}}$, we take a ±60 ms window, with $w=\mathrm{round}(0.06f_{s})$ samples and *T* denoting the window length in samples, and compute the coupling delta
  (A5) $$\Delta V_{\mathrm{saccade}}=V_{\min(T-1,t_{\mathrm{end}}+w)}-V_{\max(0,t_{\mathrm{start}}-w)}.$$
  The feature set includes its mean, standard deviation, and absolute mean across saccades.
**Excursion and vergence excursion rate.** The total vergence excursion and the normalized vergence excursion rate are
  (A6) $$E_{V}=\max_{t}V_{t}-\min_{t}V_{t},\;r_{V}=\frac{\max_{t}V_{t}-\min_{t}V_{t}}{T/f_{s}}.$$
  This rate is a descriptive feature.
**Fixation disparity spectra.** On fixation segments with at least 50 samples and cyclopean speed <30 deg/s, we compute the Welch PSD of $d_{x}$, $d_{y}$, and *V* and summarize the 1–30 Hz band. The Welch estimate uses a Hann window, segment length min(n,128), and 50% overlap. Spectral entropy is computed from the normalized band PSD:
  (A7) $$H_{\mathrm{PSD}}=-\sum_{i}p_{i}\log\left(p_{i}+\varepsilon \right),\;p_{i}=\frac{{\mathrm{PSD}}_{i}}{\sum_{j}{\mathrm{PSD}}_{j}}.$$
  Other fixation disparity spectral descriptors include dominant frequency, band power ∫PSDdf, a high/low-frequency power ratio split at the midpoint of the band (15.5 Hz for the 1–30 Hz band), and drift variability, computed as the standard deviation of the mean-removed segment and averaged over fixations.

## Appendix C. Robustness Analyses

**Table A2:** Family removal analysis for feature and fusion models.

Level	Model	Rank-1	Rank-5	EER	AUC
Feature	Monocular features	0.166	0.476	0.218	0.861
Feature	Binocular features	0.232	0.495	0.234	0.844
Feature	Mono + binocular features	0.274	0.539	0.208	0.869
Feature	Combined without disparity	0.242	0.532	0.216	0.865
Feature	Combined without vergence	0.226	0.537	0.210	0.866
Feature	Combined without fixation disparity spectra	0.282	0.553	0.211	0.870
Fusion	TCN embedding only	0.211	0.426	0.274	0.816
Fusion	Combined features only	0.274	0.539	0.208	0.869
Fusion	TCN + binocular features	0.247	0.553	0.216	0.873
Fusion	TCN + binocular features without disparity	0.226	0.524	0.216	0.872
Fusion	TCN + binocular features without vergence	0.208	0.439	0.239	0.842
Fusion	TCN + binocular features without fixation disparity spectra	0.232	0.518	0.208	0.873

*Note*: Values may differ slightly from those in the main text because these analyses were run separately.

**Table A3:** Quality sensitivity analysis for the primary round-1 S1→S2 subject-aggregate feature benchmark. Windows are retained only when the proportion of non-missing samples meets the valid-sample threshold. Metrics are Rank-1/AUC.

Valid Threshold	Classifier	Windows	Subjects	S1/S2 Rows	Monocular	Binocular	Mono + Binocular
≥0.50	GBM	129,222	76	380/380	0.168/0.861	0.226/0.844	0.274/0.869
≥0.50	RF	129,222	76	380/380	0.211/0.870	0.255/0.890	0.295/0.892
≥0.90	GBM	124,221	76	380/377	0.194/0.854	0.207/0.853	0.247/0.874
≥0.90	RF	124,221	76	380/377	0.207/0.866	0.249/0.882	0.305/0.890
≥0.95	GBM	114,897	76	378/373	0.182/0.856	0.196/0.842	0.255/0.869
≥0.95	RF	114,897	76	378/373	0.190/0.853	0.241/0.874	0.300/0.885

**Table A4:** Quality sensitivity for the combined feature set across aggregation protocols and split modes. Metrics are Rank-1/AUC. For the window-level protocol, Rank-1 is computed after averaging posteriors within subject-task-session groups, matching the grouped decision reported in the main benchmark.

Valid Threshold	Protocol/Split	Classifier	Windows	Test Rows/Groups	Rank-1/AUC
≥0.50	Subject mean/std, S1→S2	GBM	129,222	380/–	0.274/0.869
≥0.50	Subject mean/std, S1→S2	RF	129,222	380/–	0.295/0.892
≥0.50	Subject mean/std, S1 task holdout	GBM	129,222	76/–	0.066/0.839
≥0.50	Subject mean/std, S1 task holdout	RF	129,222	76/–	0.105/0.877
≥0.50	Window grouped, S1→S2	GBM	129,222	12,038/380	0.403/0.861
≥0.50	Window grouped, S1→S2	RF	129,222	12,038/380	0.334/0.862
≥0.50	Window grouped, S1 task holdout	GBM	129,222	1698/76	0.132/0.810
≥0.50	Window grouped, S1 task holdout	RF	129,222	1698/76	0.079/0.783
≥0.90	Subject mean/std, S1→S2	GBM	124,221	377/–	0.247/0.874
≥0.90	Subject mean/std, S1→S2	RF	124,221	377/–	0.305/0.890
≥0.90	Subject mean/std, S1 task holdout	GBM	124,221	76/–	0.105/0.849
≥0.90	Subject mean/std, S1 task holdout	RF	124,221	76/–	0.118/0.853
≥0.90	Window grouped, S1→S2	GBM	124,221	11,415/377	0.393/0.858
≥0.90	Window grouped, S1→S2	RF	124,221	11,415/377	0.342/0.861
≥0.90	Window grouped, S1 task holdout	GBM	124,221	1685/76	0.145/0.806
≥0.90	Window grouped, S1 task holdout	RF	124,221	1685/76	0.105/0.789
≥0.95	Subject mean/std, S1→S2	GBM	114,897	373/–	0.255/0.869
≥0.95	Subject mean/std, S1→S2	RF	114,897	373/–	0.300/0.885
≥0.95	Subject mean/std, S1 task holdout	GBM	114,897	76/–	0.092/0.845
≥0.95	Subject mean/std, S1 task holdout	RF	114,897	76/–	0.079/0.866
≥0.95	Window grouped, S1→S2	GBM	114,897	10,388/373	0.381/0.854
≥0.95	Window grouped, S1→S2	RF	114,897	10,388/373	0.332/0.856
≥0.95	Window grouped, S1 task holdout	GBM	114,897	1592/76	0.145/0.800
≥0.95	Window grouped, S1 task holdout	RF	114,897	1592/76	0.079/0.778

**Table A5:** Quality sensitivity of task transfer for the combined feature set. Each row averages the 5 matched-task cells and the 20 off-diagonal cross-task cells in the S1 task to S2 task matrix. Metrics are mean Rank-1/AUC across cells.

Valid Threshold	Classifier	Matched Cells	Cross-Task Cells	Matched vs. Cross-Task Rank-1/AUC
≥0.50	GBM	5	20	0.179/0.810 vs. 0.061/0.691
≥0.50	RF	5	20	0.253/0.860 vs. 0.086/0.720
≥0.90	GBM	5	20	0.178/0.819 vs. 0.058/0.685
≥0.90	RF	5	20	0.246/0.857 vs. 0.082/0.719
≥0.95	GBM	5	20	0.171/0.797 vs. 0.057/0.679
≥0.95	RF	5	20	0.219/0.839 vs. 0.071/0.709

**Table A6:** Test–retest reliability of the oculomotor features within each task. The table reports the mean intraclass correlation coefficient in the ICC(1,1) form across subjects between the two round 1 sessions, S1 and S2, of the same task, averaged over features and the five tasks. “Frac. ICC ≥ 0.5” is the share of feature-by-task cells reaching at least moderate reliability.

Feature Family	Mean ICC	Median ICC	Frac. ICC ≥ 0.5
Monocular	0.56	0.57	0.68
Disparity	0.30	0.29	0.11
Vergence dynamics	0.55	0.57	0.53
Fixation disparity spectra	0.46	0.54	0.54

## Appendix D. Learned-Representation Probes and Model Configuration

**Table A7:** Linear probe $R^{2}$ predicting interpretable feature families from each frozen TCN embedding, using window features, a split by subject, and RidgeCV with multiple outputs. A row-shuffled embedding control gives $R^{2}\approx 0$.

Embedding	Monocular	Disparity	Vergence	Fixation Disparity
TCN left eye	0.201	0.211	0.242	0.039
TCN raw binocular	0.138	0.416	0.156	−0.004
TCN derived binocular	0.156	0.387	0.186	0.001

**Table A8:** TCN sequence architecture and training configuration. The input views share identical architecture and training, differing only in input channels. The matched-input control duplicates the left eye to four channels, matching the raw binocular input width.

Component	Setting
Encoder	Conv1d(input_dim, 64, kernel_size = 9, padding = 4)
	BatchNorm1d(64)
	GELU
Residual blocks	4× ResidualTCNBlock(64, dilation = *d*)
	d∈{1,2,4,8}
	Conv1d(64, 64, kernel_size = 7, dilation = *d*)
	BatchNorm + GELU + Dropout(0.1) in each
	Skip connection around both convolutions
Projection	Linear(64, 64) + LayerNorm(64)
Classifier	Linear(64, $n_{\mathrm{classes}}$)
**Training **	
Optimizer	AdamW
Learning rate	0.0008
Weight decay	0.0001
Batch size	64
Epochs	8; early stopping patience = 2
Validation split	20% of S1, subject-stratified
Loss	CrossEntropy
Random state	20260517
**Input views**	
Left eye mono	Channels 0,1; L*_x_*, L*_y_*; *input_dim* = 2
Left eye equal input control	Channels 0,1,0,1; duplicated L*_x_*, L*_y_*; *input_dim* = 4
Raw binocular	Channels 0–3; L*_x_*, L*_y_*, R*_x_*, R*_y_*; *input_dim* = 4
Derived binocular	Channels 0–7; L, R plus d*_x_*, d*_y_*, V, and missingness mask *m*; *input_dim* = 8

## References

[1] Holland C., Komogortsev O.V. (2011). Biometric identification via eye movement scanpaths in reading. Proceedings of the 2011 International Joint Conference on Biometrics (IJCB), Washington, DC, USA, 11–13 October 2011.

[2] Komogortsev O.V., Jayarathna S., Aragon C.R., Mechehoul M. (2010). Biometric identification via an oculomotor plant mathematical model. Proceedings of the 2010 Symposium on Eye-Tracking Research & Applications (ETRA), Austin, TX, USA, 22–24 March 2010.

[3] Komogortsev O.V., Karpov A., Price L.R., Aragon C. (2012). Biometric authentication via oculomotor plant characteristics. Proceedings of the International Conference on Biometrics (ICB), New Delhi, India, 29 March–1 April 2012.

[4] Holland C., Komogortsev O.V. (2013). Complex eye movement pattern biometrics: Analyzing fixations and saccades. Proceedings of the International Conference on Biometrics (ICB), Madrid, Spain, 4–7 June 2013.

[5] Rigas I., Komogortsev O.V., Shadmehr R. (2016). Biometric recognition via eye movements: Saccadic vigor and acceleration cues. ACM Trans. Appl. Percept..

[6] Rigas I., Friedman L., Komogortsev O.V. (2018). Study of an extensive set of eye movement features: Extraction methods and statistical analysis. J. Eye Mov. Res..

[7] Lohr D., Griffith H.K., Komogortsev O.V. (2022). Eye Know You: Metric learning for end-to-end biometric authentication using eye movements from a longitudinal dataset. IEEE Trans. Biom. Behav. Identity Sci..

[8] Lohr D., Komogortsev O.V. (2022). Eye Know You Too: Toward viable end-to-end eye movement biometrics for user authentication. IEEE Trans. Inf. Forensics Secur..

[9] Raju M.H., Komogortsev O.V. (2025). Eye movement biometrics in virtual reality: A comparison between VR headset and high-end eye-tracker collected dataset. Proceedings of the 2025 IEEE Security and Privacy Workshops (SPW), San Francisco, CA, USA, 15 May 2025.

[10] Grandhi S.G., Samet S. (2025). Evaluating the long-term viability of eye-tracking for continuous authentication in virtual reality. Proceedings of the AI, Machine Learning and Applications, Chennai, India, 15–16 February 2025.

[11] Aziz H., Raju M.H., Komogortsev O.V. (2026). Enhancing eye movement biometrics for user authentication via continuous gaze offset score fusion. arXiv.

[12] Schor C.M. (1979). The relationship between fusional vergence eye movements and fixation disparity. Vis. Res..

[13] Jaschinski W. (2017). Individual objective and subjective fixation disparity in near vision. PLoS ONE.

[14] Bruce A.S., Atchison D.A., Bhoola H. (1995). Accommodation-convergence relationships and age. Investig. Ophthalmol. Vis. Sci..

[15] Fukushima T., Torii M., Ukai K., Wolffsohn J.S., Gilmartin B. (2009). The relationship between CA/C ratio and individual differences in dynamic accommodative responses while viewing stereoscopic images. J. Vis..

[16] Semmlow J.L., Hung G.K., Horng J.L., Ciuffreda K.J. (1994). Disparity vergence eye movements exhibit preprogrammed motor control. Vis. Res..

[17] Hung G.K., Zhu H., Ciuffreda K.J. (1997). Convergence and divergence exhibit different response characteristics to symmetric stimuli. Vis. Res..

[18] Morse S.E., Jiang B.C. (1999). Oculomotor function after virtual reality use differentiates symptomatic from asymptomatic individuals. Optom. Vis. Sci..

[19] Chen H., Zheng F., Hou F., Chen B., Huang P., Wang Y. (2021). The effect of virtual reality gaming on vergence dynamics and electroencephalogram. SID Symp. Dig. Tech. Pap..

[20] Balaban C.D., Nayak N., Williams E., Kiderman A., Hoffer M.E. (2023). Frequency dependence of coordinated pupil and eye movements for binocular disparity tracking. Front. Neurol..

[21] Lohr D., Aziz S., Friedman L., Komogortsev O.V. (2023). GazeBaseVR, a large-scale, longitudinal, binocular eye-tracking dataset collected in virtual reality. Sci. Data.

[22] Komogortsev O.V., Holland C., Jayarathna S., Karpov A. (2013). 2D linear oculomotor plant mathematical model. ACM Trans. Appl. Percept..

[23] Komogortsev O.V., Holland C.D., Karpov A., Price L.R. (2014). Biometrics via oculomotor plant characteristics: Impact of parameters in oculomotor plant model. ACM Trans. Appl. Percept..

[24] Komogortsev O.V., Karpov A., Holland C.D. (2016). Oculomotor plant characteristics: The effects of environment and stimulus. IEEE Trans. Inf. Forensics Secur..

[25] Reppert T.R., Rigas I., Herzfeld D.J., Sedaghat-Nejad E., Komogortsev O.V., Shadmehr R. (2018). Movement vigor as a traitlike attribute of individuality. J. Neurophysiol..

[26] Raju M.H., Friedman L., Lohr D., Komogortsev O.V. (2024). Signal vs noise in eye-tracking data: Biometric implications and identity information across frequencies. Proceedings of the ACM Symposium on Eye Tracking Research and Applications (ETRA), Glasgow, UK, 4–7 June 2024.

[27] Griffith H.K., Katrychuk D., Raju M.H., Aziz S., Lohr D., Komogortsev O.V. (2025). The percentile-matching technique for synthetic eye tracking signal degradation: A biometric case study. PLoS ONE.

[28] Raju M.H., Friedman L., Lohr D., Komogortsev O.V. (2024). Temporal persistence and intercorrelation of embeddings learned by an end-to-end deep learning eye movement-driven biometrics pipeline. arXiv.

[29] Raju M.H., Friedman L., Lohr D., Komogortsev O.V. (2025). From features to embeddings: Extending the temporal-persistence principle to deep-learning eye-movement biometric. Proceedings of the 2025 IEEE International Joint Conference on Biometrics (IJCB), Osaka, Japan, 8–11 September 2025.

[30] Raju M.H., Friedman L., Komogortsev O.V. (2026). Embedding reliability as a predictor of biometric performance in eye-movement biometrics. IEEE Trans. Biom. Behav. Identity Sci..

[31] Krakowczyk D.G., Prasse P., Reich D.R., Lapuschkin S., Scheffer T., Jäger L.A. (2023). Bridging the gap: Gaze events as interpretable concepts to explain deep neural sequence models. Proceedings of the ACM Symposium on Eye Tracking Research and Applications (ETRA), Tübingen, Germany, 30 May–2 June 2023.

[32] Darwish A., Pasquier M. (2013). Biometric identification using the dynamic features of the eyes. Proceedings of the 6th IEEE International Conference on Biometrics: Theory, Applications and Systems (BTAS), Washington, DC, USA, 29 September–2 October 2013.

[33] Melnyk K., Friedman L., Katrychuk D., Komogortsev O.V. (2024). Per-subject oculomotor plant mathematical models and the reliability of their parameters. Proc. ACM Comput. Graph. Interact. Tech..

[34] Katrychuk D., Lohr D., Komogortsev O.V. (2025). Oculomotor plant mathematical model in Kalman filter form with peak velocity-based neural pulse for continuous gaze prediction. IEEE Access.

[35] Melnyk K., Friedman L., Katrychuk D., Komogortsev O.V. (2025). Gaze prediction as a function of eye movement type and individual differences. Proceedings of the 2025 Symposium on Eye Tracking Research and Applications, Tokyo, Japan, 26–29 May 2025.

[36] Makowski S., Prasse P., Reich D.R., Krakowczyk D., Jäger L.A., Scheffer T. (2021). DeepEyedentificationLive: Oculomotoric biometric identification and presentation-attack detection using deep neural networks. IEEE Trans. Biom. Behav. Identity Sci..

[37] Schor C.M. (1980). Fixation disparity: A steady-state error of disparity-induced vergence. Am. J. Optom. Physiol. Opt..

[38] Schor C.M. (1979). The influence of rapid prism adaptation upon fixation disparity. Vis. Res..

[39] Alvarez T.L., Semmlow J.L., Yuan W., Munoz P. (1999). Dynamic details of disparity convergence eye movements. Ann. Biomed. Eng..

[40] Horng J.L., Semmlow J.L., Hung G.K., Ciuffreda K.J. (1998). Dynamic asymmetries in disparity convergence eye movements. Vis. Res..

[41] Cumming B.G., Judge S.J. (1986). Disparity-induced and blur-induced convergence eye movement and accommodation in the monkey. J. Neurophysiol..

[42] Alvarez T.L., Kim E.H., Yaramothu C., Granger-Donetti B. (2017). The influence of age on adaptation of disparity vergence and phoria. Vis. Res..

[43] Jaschinski W. (1997). Fixation disparity and accommodation as a function of viewing distance and prism load. Ophthalmic Physiol. Opt..

[44] Jaschinski W. (2001). Fixation disparity and accommodation for stimuli closer and more distant than oculomotor tonic positions. Vis. Res..

[45] Jaschinski W., König M. (2006). Vergence dynamics and variability of fixation disparity in children and adults. Strabismus.

[46] Jaschinski W. (2018). Individual objective versus subjective fixation disparity as a function of forced vergence. PLoS ONE.

[47] Fogt N. (2023). Topical Review: Methodological variables in clinical and laboratory measurements of fixation disparity. Optom. Vis. Sci..

[48] Gao T.Y., Wong J., Zhou L., Black J., Turnbull P.R.K., Bex P.J., Dakin S.C. (2023). Objective estimation of fusional reserves using infrared eye tracking: The digital fusion-range test. Clin. Exp. Optom..

[49] Rovira-Gay C., Mestre C., Argilés M., Pujol J. (2024). Saccade characteristics during fusional vergence tests as a function of vergence demand. Proceedings of the ACM Symposium on Eye Tracking Research and Applications (ETRA), Glasgow, UK, 4–7 June 2024.

[50] Rovira-Gay C., Mestre C., Argilés M., Pujol J. (2026). Analysis of saccade characteristics during fusional vergence tests in normal binocular vision participants. J. Eye Mov. Res..

[51] Yaramothu C., Greenspan L.D., Scheiman M., Alvarez T.L. (2019). Vergence endurance test: A pilot study for a concussion biomarker. J. Neurotrauma.

[52] Gupta P., Murray J., Beylergil S., Jacobs J., Kilbane C., Shaikh A.G., Ghasia F.F. (2023). Objective assessment of eye alignment and disparity-driven vergence in Parkinson’s disease. Front. Aging Neurosci..

[53] Shravya, Janarthanan S., Pai V.H. (2026). Comparison of accommodation and vergence parameters in early and late-onset myopic adults. BMC Ophthalmol..

[54] Hou X., Yang X., Chen B., Liao Y. (2025). Quantitative assessment of fixational disparity using a binocular eye-tracking technique in children with strabismus. J. Eye Mov. Res..

[55] Hooge I.T.C., Hessels R.S., Nyström M. (2019). Do pupil-based binocular video eye trackers reliably measure vergence?. Vis. Res..

[56] Clay V., König P., König S.U. (2019). Eye tracking in virtual reality. J. Eye Mov. Res..

[57] Adhanom I.B., MacNeilage P., Folmer E. (2023). Eye tracking in virtual reality: A broad review of applications and challenges. Virtual Real..

[58] Liebers J., Schneegass S. (2020). Gaze-based authentication in virtual reality. Proceedings of the ACM Symposium on Eye Tracking Research and Applications (ETRA), Stuttgart, Germany, 2–5 June 2020.

[59] Ajit A., Banerjee N.K., Banerjee S. (2019). Combining pairwise feature matches from device trajectories for biometric authentication in virtual reality environments. Proceedings of the 2019 IEEE International Conference on Artificial Intelligence and Virtual Reality (AIVR), San Diego, CA, USA, 9–11 December 2019.

[60] Miller R., Banerjee N.K., Banerjee S. (2020). Within-system and cross-system behavior-based biometric authentication in virtual reality. Proceedings of the 2020 IEEE Conference on Virtual Reality and 3D User Interfaces Abstracts and Workshops (VRW), Atlanta, GA, USA, 22–26 March 2020.

[61] Komogortsev O.V., Karpov A. (2013). Liveness detection via oculomotor plant characteristics: Attack of mechanical replicas. Proceedings of the International Conference on Biometrics (ICB), Madrid, Spain, 4–7 June 2013.

[62] Komogortsev O.V., Karpov A., Holland C.D. (2012). CUE: Counterfeit-resistant usable eye movement-based authentication via oculomotor plant characteristics and complex eye movement patterns. Proceedings of the SPIE 8371, Sensing Technologies for Global Health, Military Medicine, Disaster Response, and Environmental Monitoring II, Baltimore, MD, USA, 23–27 April 2012.

[63] Komogortsev O.V., Holland C.D., Karpov A. (2014). Template aging in eye movement-driven biometrics. Proceedings of the SPIE 9075, Biometric and Surveillance Technology for Human and Activity Identification XI, Baltimore, MD, USA, 5–9 May 2014.

[64] Li M., Shivakumar A., Banerjee N.K., Banerjee S. (2025). Motion forecasting attacks on behavioral biometric authentication systems in virtual reality. Proceedings of the 31st ACM Symposium on Virtual Reality Software and Technology (VRST), Montreal, QC, Canada, 12–14 November 2025.

[65] Kotwal K., Ulucan I., Özbulak G., Selliah J., Marcel S. (2025). VRBiom: A new periocular dataset for biometric applications of head-mounted display. Electronics.

[66] Busch C. (2019). Standards for biometric presentation attack detection. Handbook of Biometric Anti-Spoofing.

[67] Chingovska I., Mohammadi A., Anjos A., Marcel S. (2019). Evaluation methodologies for biometric presentation attack detection. Handbook of Biometric Anti-Spoofing.

[68] Czajka A., Becker B. (2019). Application of dynamic features of the pupil for iris presentation attack detection. Handbook of Biometric Anti-Spoofing.

[69] Friedman L., Stern H.S., Price L.R., Komogortsev O.V. (2020). Why temporal persistence of biometric features, as assessed by the intraclass correlation coefficient, is so valuable for classification performance. Sensors.

[70] Zhang Y., Yao X., Li D. (2026). Reading to Translate or Translating to Read? Modeling Translators’ Eye Movements with Multilingual Pre-Trained Models. J. Eye Mov. Res..

